# Intercomparison of methods of coupling between convection and large‐scale circulation: 2. Comparison over nonuniform surface conditions

**DOI:** 10.1002/2015MS000570

**Published:** 2016-03-18

**Authors:** C. L. Daleu, R. S. Plant, S. J. Woolnough, S. Sessions, M. J. Herman, A. Sobel, S. Wang, D. Kim, A. Cheng, G. Bellon, P. Peyrille, F. Ferry, P. Siebesma, L. van Ulft

**Affiliations:** ^1^ Department of MeteorologyUniversity of ReadingReadingUK; ^2^ National Centre for Atmospheric ScienceDepartment of Meteorology, University of ReadingReadingUK; ^3^ Department of PhysicsNew Mexico TechSocorroNew MexicoUSA; ^4^ Department of Environmental SciencesColumbia UniversityNew YorkNew YorkUSA; ^5^ Department of Applied Physics and Applied MathematicsColumbia UniversityNew YorkNew YorkUSA; ^6^ Department of Atmospheric SciencesUniversity of WashingtonSeattleWashingtonUSA; ^7^ Climate Science Branch, NASA Langley Research CentreHamptonVirginiaUSA; ^8^ The Department of PhysicsUniversity of AucklandAucklandNew Zealand; ^9^ Meteo FranceToulouseFrance; ^10^ Royal Netherlands Meteorological InstituteDe BiltNetherlands; ^11^ Delft University of TechnologyDelftNetherlands

**Keywords:** tropical convection, large‐scale parameterized dynamics

## Abstract

As part of an international intercomparison project, the weak temperature gradient (WTG) and damped gravity wave (DGW) methods are used to parameterize large‐scale dynamics in a set of cloud‐resolving models (CRMs) and single column models (SCMs). The WTG or DGW method is implemented using a configuration that couples a model to a reference state defined with profiles obtained from the same model in radiative‐convective equilibrium. We investigated the sensitivity of each model to changes in SST, given a fixed reference state. We performed a systematic comparison of the WTG and DGW methods in different models, and a systematic comparison of the behavior of those models using the WTG method and the DGW method. The sensitivity to the SST depends on both the large‐scale parameterization method and the choice of the cloud model. In general, SCMs display a wider range of behaviors than CRMs. All CRMs using either the WTG or DGW method show an increase of precipitation with SST, while SCMs show sensitivities which are not always monotonic. CRMs using either the WTG or DGW method show a similar relationship between mean precipitation rate and column‐relative humidity, while SCMs exhibit a much wider range of behaviors. DGW simulations produce large‐scale velocity profiles which are smoother and less top‐heavy compared to those produced by the WTG simulations. These large‐scale parameterization methods provide a useful tool to identify the impact of parameterization differences on model behavior in the presence of two‐way feedback between convection and the large‐scale circulation.

## Key Points

All CRMs show a increase of precipitation with the SST
SCMs exhibit a much wider range of behavior
CRMs shows similar relationships between precipitation and column‐relative humidity

## 1 Introduction

A key issue in understanding the tropical climate and its variability is the understanding of the two‐way interaction between tropical deep convection and large‐scale tropical circulations. Numerical models which simultaneously simulate convection and large‐scale circulations are computationally expensive due to the large range of spatial scales between individual convective cells and large‐scale tropical circulations. Some examples include large‐domain, high‐resolution simulations as those conducted in projects such as Cascade [e.g., *Holloway et al*., 2012] and the global cloud‐resolving modeling using Nonhydrostatic ICosahedral Atmosphere Model [e.g., *Miura et al*., 2005].

Many single column model (SCM) and cloud‐resolving model (CRM) studies have simulated the interactions of tropical deep convection with a prescribed large‐scale flow, possibly based on idealization or experimental campaign [e.g., *Tompkins*, 2001; *Xu et al*., 2002; *Derbyshire et al*., 2004; *Petch et al*., 2006]. In such studies, the time scale characterizing changes in convection is assumed to be short compared to the time scale characterizing changes in the large‐scale flow. Simulations with predefined large‐scale flow have provided much useful insight. However, the precipitation rates produced are too much constrained due to the predefined large‐scale moisture advection [*Mapes*, 1997; *Sobel and Bretherton*, 2000] and thus, such simulations cannot be used to understand the factors that control the occurrence and intensity of tropical deep convection [*Sobel et al*., 2004]. On the other hand, in nonequilibrium conditions, there is a close link between convection and the large‐scale flow such that ignoring the feedback of convection on the large‐scale flow is not appropriate [*Mapes*, 1997; *Holloway and Neelin*, 2010; *Masunaga*, 2012].

The two‐way interaction between tropical deep convection and large‐scale tropical flow has been studied at a reasonable computational cost in both SCMs and CRMs using various forms of parameterized large‐scale dynamics. This study compares two methods of parameterized large‐scale dynamics—the weak‐temperature gradient (WTG) method and the damped gravity wave (DGW) method—in a set of CRMs and SCMs.

The WTG method derives the large‐scale vertical velocity from buoyancy anomalies. It has been applied to parameterize large‐scale tropical circulations that either consume the simulated heating and accordingly maintain zero horizontal temperature gradient [*Sobel and Bretherton*, 2000] or remove the horizontal temperature gradient over a short but nonzero time‐scale [e.g., *Raymond and Zeng*, 2005; *Sessions et al*., 2010; *Daleu et al*., 2012; *Sessions et al*., 2015]. A recent innovation of the WTG method involves spectral decomposition of heating in the vertical dimension [*Herman and Raymond*, 2014]. The DGW method derives the large‐scale vertical velocity directly from the approximated momentum equations. It has been applied to study the two‐way coupling between convection and large‐scale dynamics, with the latter being simplified to a linear gravity wave of a single horizontal wavenumber [*Kuang*, 2008, 2011; *Wang et al*., 2013; *Romps*, 2012a, 2012b; *Edman and Romps*, 2015].

In the simulations using the WTG or the DGW method, the large‐scale forcing diagnosed from the domain‐mean temperature anomalies induces a moisture source. Therefore, traditional intercomparisons with prescribed large‐scale forcing (e.g., TOGA COARE and DYNAMO) and intercomparisons in which moisture source is a relaxation to a prescribed profile [*Derbyshire et al*., 2004] are extended here to simulations in which convection within the simulated domain feeds back on the large‐scale forcing which in turns drives moisture advection. The implementation of the WTG and DGW methods has always used a configuration that couples a simulated column to a reference state [e.g., *Raymond and Zeng*, 2005; *Sobel et al*., 2007; *Sessions et al*., 2010; *Wang and Sobel*, 2011; *Kuang*, 2008, 2011; *Wang and Sobel*, 2012; *Wang et al*., 2013; *Romps*, 2012a, 2012b] until recently when *Daleu et al*. [2012] developed a new configuration that couples two simulated columns via a WTG‐derived large‐scale circulation [*Daleu et al*., 2012, 2014]. Much insight has been learned from these efforts. Unfortunately, many aspects of the large‐scale parameterization methods remain uncertain since results using these two large‐scale parameterization methods show both similarities and discrepancies in model behavior.

In order to understand the different behaviors of these large‐scale parameterization methods, this international intercomparison project—the GASS‐WTG project—was developed by the Global Energy and Water Exchanges (GEWEX) Global Atmospheric Systems Modelling Panel (GASS). The goals of this project are to develop community understanding of the WTG and DGW methods, to identify differences in behavior of SCMs compared to CRMs to inform parameterization development, and to assess the usefulness of these approaches as tools for parameterization development. In this study, we will evaluate the CRMs and SCMs by comparing the strengths of the diagnosed large‐scale forcing and the precipitation rates which result from both the model physics and the parameterized large‐scale dynamical feedback. These two‐way feedbacks between convection and the large‐scale forcing will helps us to identify weaknesses in our SCM parameterization schemes and their likely behaviors in general circulation models. However, such comparison will be helpful only if a greater consistency is obtained among CRMs than among SCMs.

In Part 1 of this study [*Daleu et al*., 2015], the aim was to understand what causes discrepancies in model behavior when surface conditions in the simulated column are identical to those of the reference state. We implemented the WTG and DGW methods in a set of CRMs and SCMs. For each model, the reference state was defined from profiles obtained in the radiative‐convective equilibrium (RCE) simulation of that model. WTG and DGW simulations were performed with the same SST as in the reference state and were initialized with profiles from the reference state. Some models produced an equilibrium state which was almost identical to the corresponding RCE reference state. In contrast, other models developed a large‐scale circulation which resulted in either substantially higher or lower precipitation rates in the simulated column compared to the implied value for the RCE reference column. We also explored the sensitivity of the final equilibrium state to the initial moisture conditions. We found that while some models are not sensitive to the initial moisture conditions (independent of the method used to parameterize the large‐scale circulation), other models may support two distinct precipitating equilibrium states using either the DGW or WTG method. We also found that some models using the WTG method (but not using the DGW method) can support either an equilibrium state with persistent, precipitating convection, or an equilibrium state with zero precipitation.

*Daleu et al*. [2015] revealed some weaknesses of the WTG method. For instance, over uniform SST, the existence of the nonprecipitating equilibrium state in some models was sensitive to the choice of the parameters used in the WTG calculations (e.g., the nominal boundary layer depth). In addition, DGW simulations over uniform SST and with nearly uniform radiative forcing were more likely to reproduce the RCE reference conditions and produced large‐scale pressure velocities which were smoother compared to those produced by the WTG simulations. Aside from the choice of the large‐scale parameterization method and the details of its implementation, various other factors in the convective models were important for the evolution of convection and its interactions with parameterized large‐scale dynamics. For instance, we found that CRMs using either the WTG or DGW method produced broadly similar results, while SCMs produced a much wider range of behaviors.

Whilst *Daleu et al*. [2015] considered the case where the simulated column had the same SST as the RCE reference state, this paper focuses on the sensitivity to the SST in the simulated column, which has been a major focus of previous studies using these approaches [e.g., *Raymond and Zeng*, 2005; *Sobel et al*., 2007; *Wang and Sobel*, 2011]. *Daleu et al*. [2015] used the term “Uniform SST” to refer to conditions in which the simulated column has the same SST as in the RCE reference state. In the present study, we use the same set of CRMs and SCMs presented in *Daleu et al*. [2015] and we use the term “Non‐uniform SST” to refer to conditions in which the simulated column has a value of SST which is different to that of the RCE reference state. For each model, we fix the reference state and perform a series of WTG and DGW simulations with a range of SSTs in the simulated column. We perform a systematic comparison of the WTG and DGW methods with a consistent implementation in the models, and also a systematic comparison of the behavior of the models given the same large‐scale parameterization method.

This paper is organized as follows. Section 1 briefly describes the models that have contributed to this study. Section 2.1 outlines our implementation of the WTG and DGW methods (full details are available in *Daleu et al*. [2015]), while section 2.2 describes the configurations of our numerical simulations. Section 3 compares the results of the WTG and DGW simulations over nonuniform SSTs. Finally, the conclusions and implications of our study are discussed in section 4.

### 1.1 Description of Models

Six groups participating in this intercomparison study performed simulations with the same set of CRMs and SCMs presented in *Daleu et al*. [2015]. The models are listed in Tables 1 and 2 for CRMs and SCMs, respectively.

**Table 1:** List of Cloud‐Resolving Models (CRMs) That Participated in This Study^a^

Model type	Cloud‐Resolving Models (CRMs)				
Modeling Group	Columbia University	CNRM‐GAME	NASA	New Mexico Tech	UK Met Office
Model ID	WRF	MesoNH	LaRC‐CRM	NMTCMv3	LEMv2.4
Symbol	•	▲	♦	■	▼
Dimension	3‐D	3‐D	2‐D	2‐D	2‐D
Hor. size (km)	190 × 190	150 × 150	256	200	128
Hor. res (km)	2 × 2	3 × 3	4	1	0.5
*P_Ref_* (mm d^−1^)	4.71	4.63	4.60	4.35	4.82

a The symbols serve as a legend for results presented in section 3. *P_Ref_* is the mean precipitation rate obtained in the radiative‐convective equilibrium simulation of each CRM with an SST of 300 K.

**Table 2:** List of Single‐Column Models (SCMs) That Participated in This Study^a^

Model type	Single‐Column Models (SCMs)						
	LMD/IPSL		NASA	CNRM‐GAME	UK Met	Koninklikj Nederlands	
Modeling group					Office	Meteorologisch Insituut	
Model ID	LMDzA	LMDzB	GISS‐SCM	ARPEGEv6	UMv7.8	EC‐Earthv1	EC‐Earthv3
				(ARPv6)			
Symbol	◃	▹	○	▽	⋆	⋄	□
*P_Ref_* (mm d^−1^)	4.38	4.39	4.58	3.71	4.76	4.53	4.15

a The symbols serve as a legend for results presented in section 3. *P_Ref_* is the mean precipitation rate obtained in the radiative‐convective equilibrium simulation of each SCM with an SST of 300 K.

#### 1.1.1 Cloud‐Resolving Models

There are five CRMs, including two in three‐dimensions (3‐D) and three in two‐dimensions (2‐D). The 3‐D CRMs are the Weather Research and Forecast model version 3.3 (WRF) [*Skamarock et al*., 2008] and the mesoscale, nonhydrostatic atmospheric model (MesoNH) [*Lafore et al*., 1997]. The 2‐D CRMs are the Langley Research Center Cloud‐Resolving Model (LaRC‐CRM) [*Cheng and Xu*, 2006], the New Mexico Tech cloud model version 3 (NMTCMv3) introduced in *Raymond and Zeng* [2005], with modifications and enhancements described in *Herman and Raymond* [2014], and the Met Office Large Eddy Model at version 2.4 (LEMv2.4) [*Shutts and Gray*, 1994; *Petch and Gray*, 2001]. The reader is referred to *Daleu et al*. [2015] for a more complete description of these CRMs.

#### 1.1.2 Single‐Column Models

Two pairs of the SCMs come from different versions of the same model. One of the pairs, LMDzA and LMDzB, are the SCM versions of the atmospheric components of IPSL‐CM5A and IPSL‐CM5B [*Dufresne et al*., 2013]. The other pair, EC‐Earthv1 and EC‐Earthv3, are SCMs based on the atmospheric general circulation model IFS, cycles 31r1 and 36r4, respectively, of the European Centre for Medium‐Range Weather Forecasts (ECMWF) [*Hazeleger et al*., 2010]. ARPv6 is the SCM version of the atmospheric component of the CNRM‐CM, an updated version from that used in CMIP5 [*Voldoire et al*., 2013], GISS‐SCM is the SCM version of the National Aeronautics and Space Administration Goddard Institute for Space Studies, an updated version from that used in CMIP5 [*Schmidt et al*., 2014], and UMv7.8 is the SCM version of the UK Met Office Unified Model [*Davies et al*., 2005]. The reader is referred to *Daleu et al*. [2015] for a more complete description of these SCMs.

#### 1.1.3 Overall Approach

The CRMs have horizontal domain sizes ranging between 128 and 256 km and horizontal resolution ranging between 0.5 and 4 km. The lateral boundary conditions are periodic for all prognostic variables in all CRMs. For CRMs in 2‐D, the domain‐mean wind speeds in the along‐domain direction and in the across‐domain direction are relaxed toward vertically uniform values of 0 and 5 m s^−1^, respectively, both with a relaxation time scale of 6 h. For fair comparison of 2‐D CRM simulations with 3‐D CRM simulations and with SCM simulations, the horizontal domain‐mean wind speed components in the 3‐D CRMs and SCMs are also relaxed toward vertically uniform values of 0 and 5 m s^−1^.

For all of these models, the lower boundary condition is a spatially uniform and time‐independent SST, and the Coriolis force is zero. We force each model with the idealized cooling profile defined in *Daleu et al*. [2015]. The tendency of temperature due to radiative cooling, ${\left(\partial T/\partial t\right)}_{RC}$, is homogeneous and noninteractive throughout most of the troposphere, and it acts to maintain the temperature toward a fixed value of 200 K at levels with $\bar{p}<100$ hPa, with a relaxation time scale $\alpha_{T}^{-1}=1$ day. That is,

(1) $${\left(\frac{\partial T}{\partial t}\right)}_{RC}=\{\begin{array}{ll} -1.5 & \text{if}\;\bar{p}\geq 200\\ -1.5\left(\frac{\bar{p}-100}{100}\right)-\alpha_{T}\left(\frac{200-\bar{p}}{100}\right)(\bar{T}-200) & \text{if}\;\;100<\bar{p}<200.\\ -\alpha_{T}(\bar{T}-200) & \text{if}\;\bar{p}\leq 100 \end{array}$$

## 2 Parameterization of the Large‐Scale Dynamics and Experiment Setup

### 2.1 Parameterization of the Large‐Scale Dynamics

In the present study, the large‐scale circulation is parameterized using two methods: the WTG and DGW methods. As in *Daleu et al*. [2015], the implementation of the WTG or DGW method involves an interactive column that is coupled to a reference state.

A full description of the implementation of the WTG method is given in *Daleu et al*. [2015]. The large‐scale pressure velocity, $\bar{\omega }$ between 850 and 100 hPa acts to reduce the difference in the domain‐mean virtual potential temperature between the simulated column and the reference state, ${\bar{\theta }}_{v}-{\bar{\theta }}_{v}^{Ref}$, over a specified time‐scale, *τ*. That is,

(2) $$\bar{\omega }\frac{\partial {\bar{\theta }}_{v}^{Ref}}{\partial p}=\frac{{\bar{\theta }}_{v}-{\bar{\theta }}_{v}^{Ref}}{\tau }.$$

Above 100 hPa $\bar{\omega }$ is set to zero. Below the nominal boundary layer top, 850 hPa, we calculate the values of $\bar{\omega }$ by linear interpolation in pressure from the value diagnosed at the first model level above 850 hPa to zero at the surface. Experiments to assess sensitivities of the final equilibrium state to the depth of the boundary layer are presented in *Daleu et al*. [2015].

A full description of the implementation of the DGW method is given in *Daleu et al*. [2015]. The second‐order derivative of $\bar{\omega }$ is related to the difference in the domain‐mean virtual temperature between the simulated column and the reference state, $\bar{T_{v}}-{\bar{T}}_{v}^{Ref}$, as

(3) $$\frac{\partial }{\partial p}\left(\epsilon \frac{\partial \bar{\omega }}{\partial p}\right)=\frac{k^{2}R_{d}}{{\bar{p}}^{Ref}}({\bar{T}}_{v}-{\bar{T}}_{v}^{Ref}),$$

where *R_d_* is the gas constant of dry air. *ϵ* and *k* are the mechanical damping coefficient and the horizontal wavenumber, respectively.

As in *Daleu et al*. [2015], the large‐scale circulation parameterized using either equation (2) or (3) introduces additional source and sink terms to the potential temperature and water vapor equations only. The prognostic equation for potential temperature includes the tendency due to vertical advection by the parameterized large‐scale circulation. That is,

(4) $${\left(\frac{\partial \theta }{\partial t}\right)}_{LS}=-\bar{\omega }\frac{\partial \bar{\theta }}{\partial p}.$$

The prognostic equation for specific humidity of water vapor (*q_v_*) also includes the large‐scale tendency due to vertical advection, as well as an additional contribution representing the horizontal advection of the reference state air into the simulated domain by the parameterized large‐scale circulation. That is,

(5) $${\left(\frac{\partial q_{v}}{\partial t}\right)}_{LS}=-\bar{\omega }\frac{\partial {\bar{q}}_{v}}{\partial p}+\max\left(\frac{\partial \bar{\omega }}{\partial p},0\right)({\bar{q}}_{v}^{Ref}-{\bar{q}}_{v}).$$

### 2.2 Experiment Setup

For each model, a radiative‐convective equilibrium (RCE) simulation (no large‐scale parameterized dynamics) is first performed over an SST of 300 K. The mean thermodynamic profiles at equilibrium in that simulation are used to define the reference state of that model. We keep the reference state fixed and investigate the sensitivity of the final equilibrium state to the SST in the simulated column as in *Wang and Sobel* [2011].

For each of the models listed in Tables 1 and 2, we performed the WTG and DGW simulations of a colder column (using SSTs of 298 and 299.5 K), a warmer column (using SSTs of 300.5, 301, 301.5 and 302 K), and over a uniform SST (using an SST of 300 K; results presented in *Daleu et al*. [2015]). The adjustment time scale used in the WTG calculations is *τ* = 3 h. In the DGW calculations, we fix the value of *ϵ* to 1 d^−1^ and solve equation (3) with a single horizontal wavenumber *k* = 10^−6^ m^−1^. These are typical values used in previous WTG and DGW studies [e.g., *Herman and Raymond*, 2014; *Daleu et al*., 2012; *Wang and Sobel*, 2011; *Wang et al*., 2013], including *Daleu et al*. [2015]. They have been chosen such that the WTG simulation and the corresponding DGW simulation produce large‐scale circulations that are comparable in strength for similar temperature anomalies. The calculations of $\bar{\omega }$ given by equations (2) and (3) are performed either every 10 min (for models with integration time steps smaller or equal to 10 min) or at every model time step (for models with integration time steps greater than 10 min).

The results presented in *Daleu et al*. [2015], and in other previous studies [e.g., *Sobel et al*., 2007; *Sessions et al*., 2010] show that some SCMs and CRMs using the WTG method can sustain either a dry equilibrium state or a precipitating equilibrium state, given sufficiently different initial moisture conditions (known as multiple equilibria). Therefore, it is possible that some of our WTG simulations that exhibit precipitating equilibrium states would instead result in dry equilibrium states if initialized with very dry moisture conditions. Multiple equilibria and their dependence on parameters in the WTG calculations have already been investigated in *Daleu et al*. [2015], and they are outside the scope of the present paper.

The WTG and DGW calculations are initialized with profiles from the models' RCE reference state at 300 K and are allowed to evolve until a new quasi‐equilibrium state with parameterized large‐scale circulation is reached. The RCE reference profiles differ from model to model, with large differences obtained among SCMs [see *Daleu et al*., 2015, Figure 3]. The value of surface sensible heat flux also differs between models (not shown) but is much smaller than surface latent heat flux, such that the main balance in the RCE state is between the precipitation rate and the column‐integrated radiative cooling rate. Due to the dependence of radiative cooling profile on temperature above 200 hPa (see equation (1)), the value of column‐integrated radiative cooling rate differs from model to model. The values of mean precipitation rate obtained in the RCE simulations with an SST of 300 K are summarized in the last rows of Tables 1 and 2 for CRMs and SCMs, respectively.

We conducted a set of WTG and DGW simulations over nonuniform SSTs using each of the models listed in Tables 1 and 2. The simulations are integrated over different periods of time ranging between 50 and 250 days, as the time scale of adjustment to a quasi‐equilibrium state with the parameterized large‐scale circulation differs from model to model and also depends on which large‐scale parameterization method is used. The quasi‐equilibrium state is reached when a statistically steady state temperature and humidity profiles are achieved when averaged over a long period of time. The mean states and statistics at equilibrium of the simulations to be discussed have been obtained by averaging over the last 20 days in 50 day simulations, 30 days in 100 day simulations, and 100 days in 250 day simulations.

## 3 Results

In this section, we present the profiles of large‐scale pressure velocity and the mean precipitation rates at equilibrium for different values of SST in the simulated column. We also present the mean precipitation rates, circulation strength, and column‐relative humidity in a set of scatter plots.

### 3.1 Parameterized Large‐Scale Circulation and Mean Precipitation Rates

**Figure 1:**
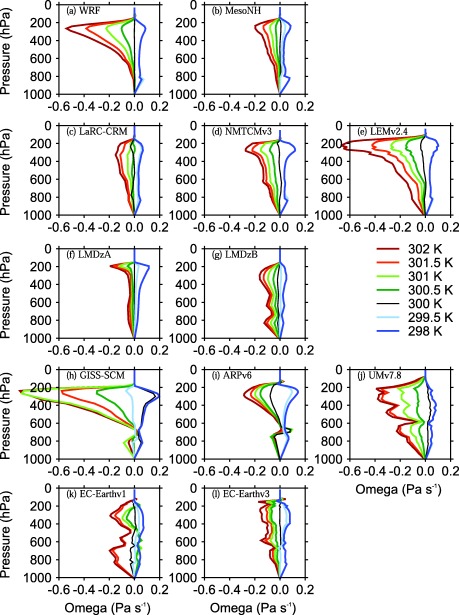
Large‐scale pressure velocities obtained at equilibrium in the WTG simulations with an SST of 298 K (dark blue), 299.5 K (light blue), 300 K (black), 300.5 K (dark green), 301 K (light green), 301.5 K (orange), and 302 K (red). Results are shown for the (a, b, c, d, and e) CRMs and (f, g, h, i, j, k, and l) SCMs. For each model, the reference profiles are their own RCE profiles at 300 K.

**Figure 2:**
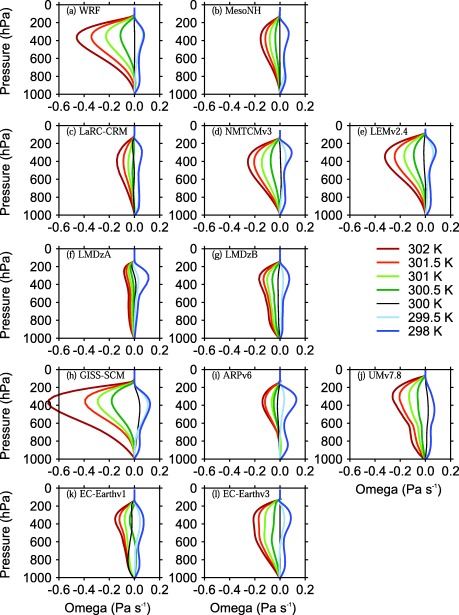
As in Figure 1, but for the equilibrium in the DGW simulations.

Figures 1 and 2 show the profiles of $\bar{\omega }$ obtained at equilibrium in the WTG and DGW simulations, respectively. Results are shown for all models listed in Tables 1 and 2 and for SSTs of 298, 299.5, 300 K (uniform SST; results presented in *Daleu et al*. [2015]), 300.5, 301, 301.5, and 302 K. For models in height coordinates, we expressed the large‐scale vertical velocities in Pa s^−1^ by applying the factor “–*ρg*,” where *ρ* is density and *g* is the gravitational acceleration.

**Figure 3:**
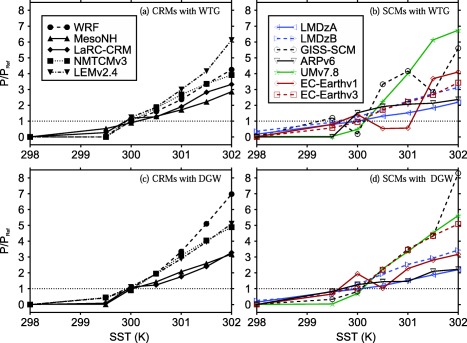
P∕P_Ref_ versus SST. The values of P are those obtained at equilibrium in the (top) WTG and (bottom) DGW simulations. Results are shown for (left) CRMs and (right) SCMs. Symbol definitions are as in Tables 1 and 2.

**Figure 4:**
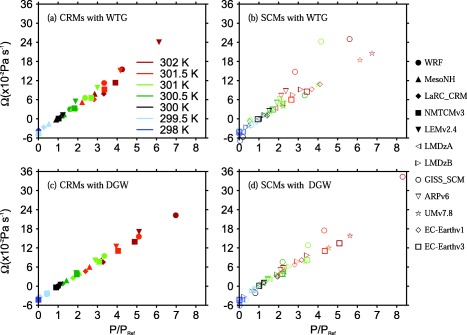
Scatter plots of Ω versus P∕P_Ref_. Results are those obtained at equilibrium in the (top) WTG and (bottom) DGW simulations with an SST of 298 K (dark blue), 299.5 K (light blue), 300 K (black), 300.5 K (dark green), 301 K (light green), 301.5 K (orange), and 302 K (red). Results are shown for (left) CRMs and (right) SCMs. Symbol definitions are as in Tables 1 and 2.

To provide a more quantitative evaluation of the WTG and DGW simulations, we calculated the ratio of mean precipitation rate in the simulated column, *P*, to the value of the corresponding RCE reference state, *P_Ref_*. We also calculated the mass‐weighted vertical integral of the large‐scale pressure velocities presented in Figures 1 and 2; $\Omega =\int \bar{\omega }dp/\Delta p$, where Δ*p* is the depth of the troposphere. The numerical values of Ω and *P*/*P_Ref_* are listed in Tables 3 and 4 for CRMs and SCMs, respectively. Figure 3 shows *P*/*P_Ref_* as a function of the SST in the simulated column, and Figure 4 shows scatter plots of Ω versus *P*/*P_Ref_* for all SSTs.

**Table 3:** Table Showing the Numerical Values of Ω (× 10^−2^ Pa s^−1^) and *P*∕*P_Ref_* for WTG and DGW Simulations With Different Values of SST in the Simulated Column^a^

Model‐CRMs	WTG or	*P*∕*P_Ref_*	SST=	SST=	SST=	SST=	SST=	SST=	SST=
	DGW	or Ω	298 K	299.5 K	300 K	300.5 K	301 K	301.5 K	302 K
		*P*∕*P_Ref_*	0.000	0.000	**1.020**	1.610	2.370	3.330	4.240
WRF	WTG	Ω	−4.210	−4.420	**0.180**	2.990	6.670	11.230	15.520
		*P*∕*P_Ref_*	0.000	0.420	**1.008**	1.950	3.350	5.100	6.970
	DGW	Ω	−4.089	−2.575	**0.110**	4.180	9.485	15.547	22.235
		*P*∕*P_Ref_*	0.002	0.529	0.896	1.303	1.714	2.220	2.857
MesoNH	WTG	Ω	−4.079	−1.577	−**0.290**	1.391	3.176	5.221	7.839
		*P*∕*P_Ref_*	0.009	0.015	**0.970**	1.425	2.073	2.594	3.190
	DGW	Ω	−3.739	−3.461	**0.060**	1.803	4.145	6.0356	8.113
		*P*∕*P_Ref_*	0.006	0.276	1.200	1.233	1.908	2.824	3.322
LaRC‐CRM	WTG	Ω	−3.549	−2.621	0.970	0.887	2.994	6.111	7.856
		*P*∕*P_Ref_*	0.000	0.084	1.102	1.233	1.757	2.394	3.282
	DGW	Ω	−3.563	−3.293	0.610	0.794	2.555	4.671	7.558
		*P*∕*P_Ref_*	0.000	0.001	**1.028**	1.830	2.679	3.352	3.912
NMTCMv3	WTG	Ω	−4.517	−4.621	**0.100**	3.303	6.570	9.221	11.317
		*P*∕*P_Ref_*	0.000	0.445	0.896	1.954	3.090	4.044	4.887
	DGW	Ω	−4.291	−2.266	−**0.388**	3.696	7.665	11.073	13.917
		*P*∕*P_Ref_*	0.000	0.000	1.240	1.886	2.997	4.159	6.124
LEMv2.4	WTG	Ω	−4.588	−4.668	1.110	5.471	9.745	15.162	24.048
		*P*∕*P_Ref_*	0.000	0.413	1.117	1.923	2.888	3.953	5.111
	DGW	Ω	−4.460	−2.658	0.464	4.129	8.103	12.436	17.031

a Results in bold correspond to $|\Omega |<0.4\times 10^{-2}$ Pa s^−1^ (or $\bar{\omega }\approx 0$) or $0.9<P/P_{Ref}<1.1$. If both Ω and *P*∕*P_Ref_* are bold, the simulation with large‐scale parameterization reproduces the RCE state to a good approximation.

**Table 4:** Same as Table 3, but Lists SCM Results

Model‐SCMs	WTG or	*P*∕*P_Ref_*	SST=	SST=	SST=	SST=	SST=	SST=	SST=
	DGW	or Ω	298 K	299.5 K	300 K	300.5 K	301 K	301.5 K	302 K
		*P*∕*P_Ref_*	0.176	0.790	**0.997**	1.313	1.520	1.829	2.192
LMDzA	WTG	Ω	−4.071	−1.037	−**0.015**	1.385	2.240	3.387	4.806
		*P*∕*P_Ref_*	0.15	0.804	**0.982**	1.187	1.530	1.874	2.201
	DGW	Ω	−4.145	−0.972	−**0.065**	0.931	2.169	3.437	4.652
		*P*∕*P_Ref_*	0.362	0.929	1.290	1.694	2.273	2.729	3.127
LMDzB	WTG	Ω	−2.670	−0.30	1.180	2.992	5.475	7.470	9.193
		*P*∕*P_Ref_*	0.248	0.638	1.269	1.922	2.537	2.940	3.437
	DGW	Ω	−3.325	−1.462	1.030	3.676	6.153	7.726	9.689
		*P*∕*P_Ref_*	0.044	1.200	0.180	3.325	4.161	2.833	5.605
GISS‐SCM	WTG	Ω	−6.888	1.100	−5.700	7.371	24.25	14.760	25.022
		*P*∕*P_Ref_*	0.021	0.330	0.820	2.201	3.498	4.319	8.296
	DGW	Ω	−6.095	−4.395	−2.180	7.566	12.837	17.475	34.335
		*P*∕*P_Ref_*	0.003	0.000	1.530	1.920	2.067	2.132	2.368
ARPv6	WTG	Ω	−5.486	−3.852	2.230	5.055	6.132	7.210	8.658
		*P*∕*P_Ref_*	0.000	0.832	1.260	1.442	1.464	2.098	2.223
	DGW	Ω	−5.853	−1.122	0.972	2.046	2.340	5.0256	5.673
		*P*∕*P_Ref_*	0.022	0.036	0.470	2.228	4.002	6.129	6.743
UMv7.8	WTG	Ω	−4.528	−4.600	−2.130	4.053	10.751	18.537	20.610
		*P*∕*P_Ref_*	0.003	0.0343	0.700	2.257	3.350	4.534	5.623
	DGW	Ω	−4.465	−4.437	−1.240	3.875	7.734	12.104	15.878
		*P*∕*P_Ref_*	0.011	0.792	1.420	0.529	0.558	3.682	4.101
EC‐Earthv1	WTG	Ω	−4.060	−1.262	0.990	−0.741	−0.192	9.275	10.855
		*P*∕*P_Ref_*	0.002	0.662	1.920	1.024	2.271	2.807	3.170
	DGW	Ω	−4.117	−1.737	2.990	0.583	4.713	6.736	8.187
		*P*∕*P_Ref_*	0.003	0.577	**0.940**	1.720	2.202	2.648	3.430
EC‐Earthv3	WTG	Ω	−4.209	−1.860	−**0.135**	2.927	4.611	6.008	8.523
		*P*∕*P_Ref_*	0.0122	0.813	**1.014**	2.191	3.448	4.344	5.087
	DGW	Ω	−4.280	−0.986	**0.146**	3.873	8.138	11.061	13.460

#### 3.1.1 Variations Between Models

For a given SST in the simulated column, the characteristic vertical structure of the large‐scale circulation at equilibrium differs from model to model, and it also depends on the large‐scale parameterization method used. Over an SST of 302 K (red curves in Figures 1 and 2), for example, models using the WTG method exhibit a range of large‐scale pressure velocity profiles which vary from unimodal ascent through the column with very top‐heavy profiles (e.g., WRF; Figure 1a), to more uniform unimodal profiles (e.g., LaRC‐CRM; Figure 1c), to bimodal profiles (e.g., EC‐Earthv1; Figure 1k), to profiles with distinct minima near the freezing level (e.g., UMv7.8; Figure 1j), including some with weak descent near the freezing level (e.g., GISS‐SCM; Figure 1h). As seen in *Daleu et al*. [2015], the DGW method produces large‐scale pressure velocity profiles which are smoother than those produced using the WTG method (compare Figures 1 and 2).

Over cold SSTs (298 and 299.5 K), some models produce large‐scale pressure velocity profiles which are insensitive to the SST. In such simulations, convection is inhibited completely and the heating due to the diagnosed large‐scale circulation balances the prescribed radiative cooling. Some examples are the WTG simulations of LEMv2.4 with SSTs of 298 and 299.5 K which produce zero precipitation rates (see Table 3) and indistinguishable large‐scale pressure velocity profiles (see dark blue and light blue curves in Figure 1e).

Over warm SSTs, the large‐scale pressure velocity profiles and precipitation rates are sensitive to the SST in all the models using either the WTG or DGW method. The sensitivity differs from model to model, and there is much diversity even among CRMs. Using the DGW method, for example, the two 3‐D CRMs (WRF and MesoNH) with an SST of 302 K produced large‐scale pressure velocities and precipitation rates which differ by more than a factor of two (compare the red curves in Figures 2a and 2b, and the values of *P*/*P_Ref_* in Table 3). However, all CRMs with an SST ≥301 K have large‐scale pressure velocities increasing upward to around 400 hPa using the DGW method and to around 250 hPa using the WTG method.

The large‐scale pressure velocity profiles produced in most SCM simulations vary considerably from the very top‐heavy profiles (e.g., GISS‐SCM using the DGW method, see Figure 2h) through weakly top‐heavy profiles (e.g., LMDzB using the WTG method, see Figure 1g) to the bottom‐heavy profiles (e.g., EC‐Earthv1 using the WTG method; see Figure 1k), and some of the pressure velocity profiles show very detailed structures in the vertical (e.g., UMv7.8 using the WTG method; see Figure 1j). Similar to the results of *Wang et al*. [2013], the pressure velocity profiles produced using the DGW method are much smoother and tend to be slightly less top‐heavy compared to those produced using the WTG method (compare Figures 1 and 2).

#### 3.1.2 Variations With SST

The impact of the SST is readily seen. At SST = 298 K, all the models using either the WTG or DGW method produce uniform large‐scale descent (see the dark blue curves in Figures 1 and 2). In some of these simulations, the large‐scale circulation inhibits precipitating convection completely (e.g., NMTCMv3 using the DGW method; see Table 3), while in others an equilibrium state with light precipitation can be achieved (e.g., LMDzB using the WTG method; see Table 4).

At SST = 299.5 K, all CRMs using either the WTG or DGW method produced uniform large‐scale descent. With the exception of GISS‐SCM using the WTG method, which produces large‐scale ascent in the upper troposphere (light blue curve in Figure 1h), the SCMs produce either a uniform large‐scale descent throughout the column (e.g., ARPv6 using the WTG method; light blue curve in Figure 1i) or large‐scale descent in the upper troposphere and a very weak circulation in the lower troposphere (e.g., EC‐Earthv3 using the WTG method; light blue curve in Figure 1l). The WTG and DGW simulations which produce uniform large‐scale descent result in very low precipitation compared to the value of the RCE reference state, consistent with the negative moisture transport implied by the resulting large‐scale circulation (e.g., MesoNH using the WTG method; see Table 3), with some simulations producing zero precipitation at equilibrium (e.g., WRF using the WTG method; see Table 3). The WTG and DGW simulations which produce large‐scale descent in the upper troposphere and a very weak circulation in the lower troposphere are dominated by shallow convection and thus, result in smaller reductions in precipitation compared to the value of the RCE reference state (e.g., EC‐Earthv3 using the WTG method; see Table 4). However, in the WTG simulation of GISS‐SCM with an SST of 299.5 K the mean precipitation rate at equilibrium is slightly increased (with respect to the value of the RCE reference state) to balance the net small cooling produced by the large‐scale ascent in the upper troposphere. In contrast, the DGW simulation of GISS‐SCM with an SST of 299.5 K produces a different sign of the circulation with a reduction of precipitation (see Table 4).

The results of the WTG and DGW simulations over uniform SST are presented in *Daleu et al*. [2015]. There, we considered that a WTG or DGW simulation over a uniform SST replicated the corresponding RCE reference state to a good approximation if $0.9<P/P_{Ref}<1.1$ and $-0.4\times 10^{-2}<\Omega <0.4\times 10^{-2}$ Pa s^−1^. The values of Ω and *P*/*P_Ref_* for such simulations are both bold‐faced in Tables 3 and 4. Some models replicate the corresponding RCE reference state to a good approximation. In contrast, other models sustain a large‐scale ascent (or descent) which results in substantially higher (or lower) precipitation rate in the simulated column compared to the value of the corresponding RCE reference state.

Those models which produce a lower precipitation rate over a uniform SST of 300 will not produce a mean precipitation rate which is equivalent to the value of the RCE reference state unless the SST in the simulated column is increased, consistent with the results of *Raymond and Zeng* [2005]. An example is UMv7.8 using the WTG method (see *P*/*P_Ref_* as a function of the SST; green curve in Figure 3b). Similarly, models which produce a higher precipitation rate will not produce a mean precipitation rate which is equivalent to the value of the RCE reference state unless the SST in the simulated column is decreased (e.g., ARPv6 using the WTG method; solid black curve in Figure 3b).

An SST of 300.5 K results in substantially higher precipitation rate ( $P/P_{Ref}>1.1$) in all the WTG and DGW simulations, except EC‐Earthv1. A large proportion of these simulations produce uniform large‐scale ascent (e.g., GISS‐SCM using the DGW method, dark green curve in Figure 2h). Other simulations produce large‐scale circulations with a layer of descent near the freezing layer, but which nonetheless result in net column‐integrated cooling and moistening of the simulated column (e.g., ARPv6 using the WTG method, dark green curve in Figure 1i and $P/P_{Ref}>1.1$ in Table 4). In contrast, the WTG and DGW simulations of EC‐Earthv1 with an SST of 300.5 K produce large‐scale circulations with ascent in the upper troposphere and descent in the lower troposphere (dark green curves in Figures 1k and 2k), despite producing ascent in the lower troposphere over a uniform SST of 300 K (black curves in Figures 1k and 2k). In this model using the DGW method, the large‐scale circulation cools and moistens the upper troposphere at the same rates as it warms and dries the lower troposphere. As a result, the column‐integrated heating and moistening rates produced by the large‐scale circulation are both negligible and thus, the simulated column achieves an equilibrium precipitation rate which is very close to the corresponding RCE reference state (see value of *P*/*P_Ref_* in Table 4). In contrast, using the WTG method, the upper tropospheric cooling and moistening do not prevent a reduction in precipitation rate due to the lower tropospheric warming and drying (see Table 4). A similar result is obtained in the WTG simulation of EC‐Earthv1 with an SST of 301 K (see the light green curve in Figure 1k and the value of *P*/*P_Ref_* in Table 4). The WTG and DGW simulations of EC‐Earthv1 with an SST of 301 K produce different signs of the integrated circulation.

At SSTs > 301 K, the mean precipitation rate is increased compared to the value of the corresponding RCE reference state in all the models using either the WTG or DGW method. These simulations produce uniform large‐scale ascent in the simulated column, with the exceptions of the WTG simulations of ARPv6 and GISS‐SCM, in which a thin layer of descent between 750 and 650 hPa does not prevent an increase in mean precipitation rate.

For all CRMs using either the WTG or the DGW method, the simulated column evolves toward a new quasi‐equilibrium state with mean precipitation rate increasing nonlinearly with SST, consistent with SCM results from *Sobel and Bretherton* [2000], and *Ramsay and Sobel* [2011]. In contrast, the SCMs show sensitivities of the mean precipitation rate to the SST which are not always monotonic (e.g., EC‐Earthv1 using either the WTG or DGW method; solid red curves in Figures 3b and 3d).

Within an individual model, the sensitivity of precipitation rate to the SST depends on which large‐scale parameterization method is used. An example is WRF which shows a stronger sensitivity under the DGW method than under the WTG method (compared the dashed curves in Figures 3a and 3c). On the other hand, given one of the large‐scale parameterization methods (either the WTG or DGW method), the sensitivity of precipitation rate to the SST differs from model to model.

An approximately linear relationship between Ω and the mean precipitation rate is expected, since the mean vertical motion and mean vertical moisture advection are correlated. In our study, despite the differences in the pressure velocity profiles, Ω and the mean precipitation rate show a fairly linear relationship (see Figure 4) and only models with unusual vertical pressure velocity profiles shows deviations from this linear relationship (e.g., GISS‐SCM using the WTG method; see Figure 1h and circles in Figure 4b). Most of the models meet the expectation that the large‐scale circulation and precipitation rate should increase with SST. Models which show a monotonic increase of precipitation with SST also show a monotonic increase of precipitation with Ω (WRF using the WTG method; dashed curve with solid circles in Figure 3a and solid circles in Figure 4a). In contrast, models which show a nonmonotonic increase of precipitation with SST also show a nonmonotonic increase of precipitation with Ω (e.g., GISS‐SCM using the WTG method at warm SSTs; dashed black curve with circles in Figure 3b and circles in Figure 4b).

### 3.2 Precipitation and Column Relative Humidity

**Figure 5:**
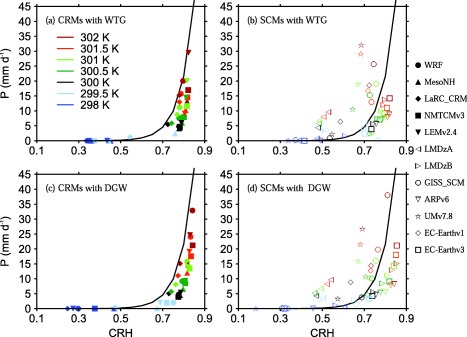
Scatter plots of P versus CRH (column relative humidity; the column‐integrated water vapor divided by its saturation value). The results are those obtained at equilibrium in the (top) WTG and (bottom) DGW simulations with an SST of 298 K (dark blue), 299.5 K (light blue), 300 K (black), 300.5 K (dark green), 301 K (light green), 301.5 K (orange), and 302 K (red). Results are shown for (left) CRMs and (right) SCMs. Symbol definitions are as in Tables 1 and 2. The solid curve is the exponential fit for the observed daily mean precipitation over the tropical oceans obtained by *Bretherton et al*. [2004].

In this section, we examine the relationship between precipitation and the column relative humidity (hereafter *CRH*) in our WTG and DGW simulations. *CRH* is calculated as the ratio of column‐integrated water vapor to its saturation value. Figure 5 shows scatter plots of *P* versus *CRH*. It also shows the exponential fit for the observed daily mean precipitation over the tropical oceans obtained by *Bretherton et al*. [2004] (solid curve). That is

(6) $$P(mmd^{-1})=\text{exp}[15.6(CRH-0.603)].$$

**Figure 6:**
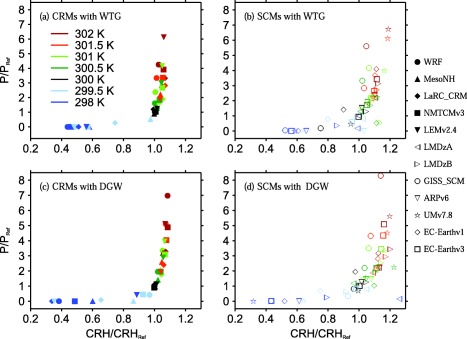
Scatter plots of P∕P_Ref_ versus CRH/CRH_Ref_, where CRH_Ref_ is the column relative humidity of the corresponding RCE reference state. The results are those obtained at equilibrium in the (top) WTG and (bottom) DGW simulations with an SST of 298 K (dark blue), 299.5 K (light blue), 300 K (black), 300.5 K (dark green), 301 K (light green), 301.5 K (orange), and 302 K (red). Results are shown for (left) CRMs and (right) SCMs. Symbol definitions are as in Tables 1 and 2.

To account for the variations in *CRH* of the RCE reference state, we also consider Figure 6, which shows scatter plots of the ratios *P*/*P_Ref_* versus *CRH*/*CRH_Ref_*, where *CRH_Ref_* is the column‐integrated relative humidity of the RCE reference state. The values of *P* and *CRH* are those obtained at equilibrium in the WTG and DGW simulations of each of the models listed in Tables 1 and 2 with the values of SST ranging between 298 and 302 K.

Generally, the mean precipitation rate increases as *CRH* increases, except in the DGW simulations of LMDzA with SSTs 6300.5 K in which *CRH* decreases while precipitation rate increases (see left‐facing triangles in Figure 5d). The decrease of *CRH* with mean precipitation rate is unusual, but we do not investigate this further in this study.

In a large proportion of the models, there is a threshold value of *CRH* below which there is virtually no precipitation or strongly reduced precipitation rate (with respect to the value of the RCE reference state) and above which precipitation rate rapidly increases with *CRH*. Below this threshold, the WTG and DGW simulations show changes in mean precipitation rate that are relatively small for large changes in *CRH*. Above this threshold, a significant increase in precipitation rate is obtained, followed by a sharp pickup of mean precipitation rate as *CRH* increases further. The value of this threshold varies from one model to another and it also depends on the large‐scale parameterization method used.

These relationships between *CRH* and mean precipitation rate are qualitatively similar to that seen in observations over the tropical ocean regions [*Bretherton et al*., 2004] (see solid curves in Figure 5), and in other idealized models [e.g., *Raymond and Zeng*, 2005; *Wang and Sobel*, 2011], but there are significant quantitative differences. For instance, CRMs using either the WTG or DGW method produce similar relationship between *P* and *CRH*. However, all CRMs using either the WTG or DGW method have a higher threshold than observations and their mean precipitation rates rise more abruptly with *CRH* than in observations (see Figures 5a and 5c). In contrast, SCMs show a much larger variety of relationships (see Figures 5b and 5d). Moreover, the transition from near zero precipitation to rapid increase in precipitation with *CRH* is sharper in some models compared to others (e.g., compare *P* versus *CRH* in the WTG simulations of UMv7.8 and LMDzB; stars and right‐facing triangles in Figure 5b, respectively). When *P* and *CRH* are scaled by their reference values (see Figure 6), the CRMs produce a relatively tight relationship. The spread among SCMs is also clearly reduced, although considerable scatter remains. In general, the threshold occurs at around *CRH_Ref_* and beyond that, *P* increases much more rapidly with *CRH* in CRMs than in SCMs.

### 3.3 Budget Analysis

As in *Daleu et al*. [2015], we analyze the budgets in order to clarify the differences among RCE, WTG, and DGW simulations. For a simulation with parameterized large‐scale circulation, the heat and moisture budgets are written as

(7) $$H+P+R+H_{LS}=0\;and\;E-P+M_{LS}=0,$$

respectively. *E*, *H*, *P*, and *R* denote the domain and time‐averaged values of surface evaporation, surface sensible heat flux, precipitation rate, and vertically integrated radiative cooling rate, respectively. The heating rate and moistening rate due to the diagnosed large‐scale circulation ( $H_{LS}=C_{p}{〈\partial \bar{T}/\partial t〉}_{LS}$ and $M_{LS}=L_{v}{〈\partial \bar{q}/\partial t〉}_{LS}$, respectively) are zero by definition for the RCE simulations. *C_p_* is the heat capacity at constant pressure and *L_v_* is the latent heat of vaporization.

**Figure 7:**
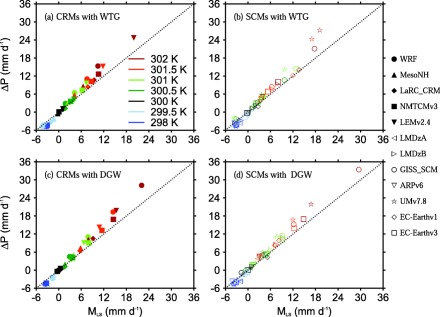
Scatter plots of ΔP versus M_LS_. The results are those obtained at equilibrium in the (top) WTG and (bottom) DGW simulations with an SST of 298 K (dark blue), 299.5 K (light blue), 300 K (black), 300.5 K (dark green), 301 K (light green), 301.5 K (orange), and 302 K (red). Results are shown for (left) CRMs and (right) SCMs. The dotted oblique line corresponds to ΔP = M_LS_. Symbol definitions are as in Tables 1 and 2.

**Figure 8:**
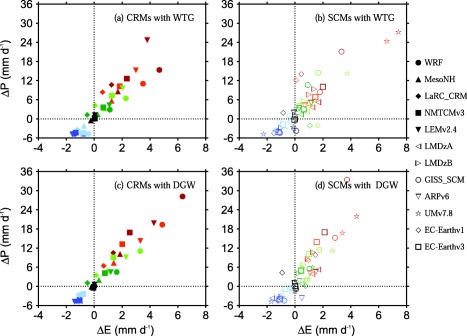
Scatter plots of ΔP versus ΔE. Results are those obtained at equilibrium in the (top) WTG and (bottom) DGW simulations over an SST of 298 K (dark blue), 299.5 K (light blue), 300 K (black), 300.5 K (dark green), 301 K (light green), 301.5 K (orange), and 302 K (red). Results are shown for (left) CRMs and (right) SCMs. Symbol definitions are as in Tables 1 and 2.

From the moisture budget equation, the changes in mean precipitation rate with respect to the value of the RCE reference state, Δ*P*, must be due to changes in surface evaporation with respect to the value of the RCE reference state, Δ*E*, and/or the moistening rate due to the large‐scale circulation *M_LS_*. Figures 7 and 8 show scatter plots of Δ*P* versus *M_LS_* and scatter plots of Δ*P* versus Δ*E*, respectively.

Both CRMs and SCMs show fairly linear relationships between Δ*P* and *M_LS_*. However, the slope is not one‐to‐one (dotted oblique line in Figure 7), which implies changes in surface evaporation as shown in Figure 8. Δ*E* increases with Δ*P* in a large proportion of the WTG and DGW simulations, and there are only a few simulations which show an enhancement of convective activity associated with a reduction in surface evaporation (e.g., WTG simulation of LaRC‐CRM an SST of 300.5 K, dark green solid diamond in Figure 8a) or which show a suppression in convective activity associated with an increase in surface evaporation (e.g., the WTG simulation of EC‐Earthv1 with SST of 300.5 K, dark green diamond in Figure 8b).

The sensitivity of surface fluxes (sum of sensible heat and latent heat fluxes) to changes in near‐surface perturbation winds due to changes in convective activity has been somewhat constrained in this study by imposing a mean horizontal wind speed in the surface flux calculations. As a result, Δ*E* is generally much smaller than Δ*P*, such that changes in precipitation are largely balanced by the large‐scale moistening rates. This is readily seen in Figures 7 and 8. For a large proportion of the simulations, the values of *M_LS_* are about or more than two third the values of Δ*P*.

We now examine the relationship between Δ*P* and the normalized gross moist stability (NGMS), Γ. Γ is defined as the dimensionless number which relates the net lateral outflow of moist static energy from a convective region to a measure of the strength of convection in that region [*Raymond et al*., 2009]. That is

(8) $$\Gamma =-〈\bar{\omega }\partial \bar{h}/\partial p〉/L_{v}〈\bar{\omega }\partial \bar{q_{v}}/\partial p〉,$$

where *h* is the moist static energy. Following *Daleu et al*. [2015],

(9) $$\Gamma =-(M_{LS}+H_{LS})/M_{LS},$$

and a diagnostic equation for Δ*P* is

(10) $$\Delta P=\frac{\Gamma +1}{\Gamma }\Delta E+\frac{\Delta H+\Delta R}{\Gamma },$$

where Δ*H* and Δ*R* are, respectively, the changes in surface sensible heat flux and column‐integrated radiative cooling rates with respect to the values of the RCE reference state. The reader is referred to *Daleu et al*. [2015] for a derivation of equation (10).

As discussed above, Δ*H* is much smaller than Δ*P*. Δ*R* is also much smaller than Δ*P* as a result of imposing a fixed radiative cooling profile throughout most of the troposphere. Also, most of these simulations show that the sum of Δ*H* and Δ*R* is much smaller than Δ*E*, such that the factor (Γ+1)/Γ largely describes the strength of the relationship between Δ*P* and Δ*E* (see equation (10)).

**Figure 9:**
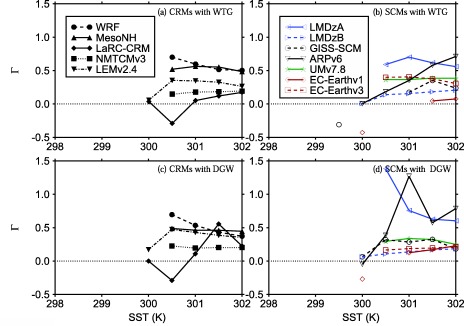
Γ versus SST. The values of Γ are those obtained at equilibrium in the (top) WTG and (bottom) DGW simulations which produce significant large‐scale ascent only (P∕P_Ref_ > 1.1 with $\Omega >0.4\times 10^{-2}$ Pa s^−1^). Results are shown for (left) CRMs and (right) SCMs. Symbol definitions are as in Tables 1 and 2.

For the WTG and DGW simulations which reproduce the RCE reference state to a good approximation, Γ is a poor diagnostic since $M_{LS}+H_{LS}$ and *M_LS_* are both close to zero, consistent with a weak large‐scale circulation. Moreover, Γ measures the efficiency of convection in removing moisture static energy from the column and thus, is not a particularly useful diagnostic when convection is strongly suppressed. Therefore, the values of Γ for the WTG and DGW simulations which result in significant large‐scale descent are not relevant, and we consider Figure 9, which shows Γ as a function of SST for the WTG and DGW simulations which result in significant large‐scale ascent only. These are simulations which produce *P*∕*P_Ref_* > 1.1 with Ω > 0.4 × 10^−2^ Pa s^−1^.

Most CRM simulations which result in significant large‐scale ascent have positive values of Γ and the WTG and DGW simulations of LaRC‐CRM with an SST of 300.5 K are the only CRM simulations which have negative values of Γ (black diamonds in Figures 9a and 9c). Among SCMs, simulations with warm SSTs which produce significant large‐scale ascent have positive values of Γ. Negative Γ in some SCMs are obtained in the simulations which result in either large‐scale ascent over a cold SST (e.g., the WTG of GISS‐SCM with an SST of 299.5 K; Table 4 and black circles in Figure 9b) or large‐scale ascent over a uniform SST (e.g., the WTG and DGW simulations of EC‐Earthv1 and the DGW simulation of ARPv6 over a uniform SST of 300 K; Table 4 and red diamonds in Figures 9b and 9d, and black down‐facing triangles in Figure 9d). In the simulations which result in significant large‐scale ascent and have negative values of Γ, *M_LS_* values are positive. Therefore, negative values of Γ are the result of a deficit of cooling over moistening rates. That implies a reduction in evaporation despite an increase in precipitation rate in those simulations (e.g., dark green diamond in Figures 8a and 8c). With the exception of the negative values of Γ, Γ generally ranges between 0 and 1, with only few SCM simulations having Γ > 1 (e.g., LMDzA using the DGW with an SST of 300.5 K; blue left‐facing triangles in Figure 9d).

CRMs (except LaRC‐CRM) and three SCMs (EC‐Earthv3, UMv7.8 and LMDzB) using either the WTG or DGW method have Γ which is relatively insensitive to the SST. In those models, Δ*E*, and hence *M_LS_* scale approximately linearly with Δ*P*. In the other four SCMs and LaRC‐CRM, Γ show large sensitivity including nonmonotonic behavior, and there are substantial differences in the relationship between Γ and SST depending on which large‐scale parameterization is used (e.g., compare Γ versus SST for the WTG and DGW simulations of ARPv6; down‐facing triangles in Figures 9b and 9d).

In this study, there is no straightforward relation between Γ and the top‐heaviness of $\bar{\omega }$ calculated as the mass‐weighted vertical integral of the pressure velocity over the layer at 500–100 hPa (see definition in section 3.1). In addition, Γ does not explain the difference between different models sensitivity to SST. Despite the fact that studies of this nature allow convection to interact with the large‐scale dynamics, there are many differences between these feedbacks compared to full General Circulation Models (GCMs) and the real tropical circulations. For instance, evaporation is not tied to the large‐scale circulation, the moisture convergence is directly tied to the dynamical convergence without any contribution from the rotational part of the flow, and radiation is noninteractive. Therefore, the NGMS in these studies may have very different characteristics compared to that of full GCMs and the real tropical circulations.

On the other hand, *Wang and Sobel* [2011] idealize horizontal moisture convergence and radiation in the same way as in our study and found that Γ is a predictor of Δ*P* in the precipitating regime. In this study, only two models exhibit positive values of Γ which are a monotonically decreasing function of SST or *P* as in *Wang and Sobel* [2011]. These models are WRF and LEMv2.4 using either the WTG or DGW method (circles and down‐facing triangles in Figures 9a and 9c). In contrast to the result of *Wang and Sobel* [2011], some models exhibit positive values of Γ which are a monotonically increasing function of SST or *P* (e.g., ARPv6 using the WTG method with warm SSTs, down‐facing triangles in Figure 9b) while other models exhibit positive or negative values of Γ which are not directly related to SST or *P* (e.g., LaRC‐CRM using the DGW method with warm SSTs, diamonds in Figure 9c). In the latter case, Γ and Δ*E* are both important to predict Δ*P* (see equation (10)).

## 4 Conclusions

In this international intercomparison project, we used the WTG and DGW methods to study the two‐way interaction between convection and large‐scale circulations in various CRMs and SCMs. Using the WTG method, we derived the large‐scale circulation that reduces the virtual potential temperature anomalies over a given time scale [*Raymond and Zeng*, 2005; *Sobel et al*., 2007; *Sessions et al*., 2010; *Daleu et al*., 2012], and using the DGW we simplified the large‐scale circulation to a linear gravity wave of a single horizontal wave number [*Kuang*, 2008, 2011; *Romps*, 2012a, 2012b]. In both cases, the derived large‐scale circulation couples a model to a reference state defined with profiles generated from previous RCE simulations of the same model. In *Daleu et al*. [2015], we analyzed WTG and DGW simulations over a uniform SST. In this paper, we kept the reference state fixed and conducted WTG and DGW simulations with different values of SST in the simulated column.

The WTG and DGW simulations with a cold (or a warm) SST result in lower (or higher) precipitation rates (compared to the value of the RCE reference state) in all CRMs and in a large proportion of the SCMs. In a few SCMs, a WTG simulation over a warm SST and a corresponding DGW simulation produce different signs of the circulation. In those SCMs, different signs of the circulation occur because the WTG simulation produces large‐scale ascent over a cold SST or large‐scale descent over a warm SST.

In general, the behavior across models for a given large‐scale parameterization method is different, and the behavior of an individual model also depends on which large‐scale parametrization is used. However, DGW simulations do produce large‐scale pressure velocity profiles which are smoother than those produced by WTG simulations, and consistent with the results of *Wang et al*. [2013], DGW simulations generally produce large‐scale pressure velocity profiles which are less top‐heavy compared to those produced by WTG simulations.

All CRMs and five out of the seven SCMs show a monotonic increase of mean precipitation rate with SST using either the WTG or DGW method. A similar relationship between precipitation rate and SST was produced in *Sobel and Bretherton* [2000] and *Ramsay and Sobel* [2011]. The other two SCMs show sensitivity of the mean precipitation rate with SST which is not always monotonic. CRMs show a fairly linear relationship between mean precipitation rate and the amplitude of the diagnosed vertically integrated large‐scale circulation, while a few SCMs show deviations from this linear relationship, particularly for simulations with warm SST.

Precipitation is an increasing function of the column relative humidity, with the former increasing rapidly as the latter passes a threshold. A similar relationship is found in other numerical modeling studies [*Wang and Sobel*, 2011; *Raymond and Zeng*, 2005], and is consistent with observations [*Bretherton et al*., 2004; *Holloway and Neelin*, 2009]. All CRMs using either the WTG or DGW method show a similar relationship between mean precipitation rate and column‐relative humidity. They are all moister and the resulting mean precipitation rate increases more abruptly with column relative humidity than in observations. SCMs show a much wider range of relationships between precipitation rate and column‐relative humidity, although this spread is reduced when values are normalized by their RCE values.

In our WTG and DGW simulations, the change in precipitation with respect to the value of the RCE reference column is largely balanced by the moistening rate due to the large‐scale circulation. We calculated the NGMS for simulations with significant large‐scale ascent at equilibrium. A large proportion of those simulations exhibited positive values of NGMS, ranging between 0 and 1, and only a few simulations exhibit negative values of NGMS or values of NGMS that approach 1.5. Those which exhibit negative values of NGMS have a deficit of cooling over moistening rates, which implies a reduction in evaporation despite an increase in precipitation rate. Most CRMs and three SCMs using either the WTG or DGW method show small sensitivity of the NGMS with the SST. In the other CRM and the other four SCMs, the relationship between NGMS and SST varies considerably and depends on the large‐scale parameterization method used. In this study, Γ is not related to the shape of the large‐scale pressure velocity profile and does not explain the difference between different model's sensitivity to SST. That is, in comparison to real tropical circulations, the NGMS in this configuration may not be a very important diagnostic due to the way in which evaporation, horizontal moisture convergence and radiation are idealized.

In this intercomparison, project convection feeds back on the large‐scale forcing, the moisture source is induced by the derived large‐scale motion, and the precipitation rate produced is the result of both the model physics and parametrized large‐scale dynamical feedback. Therefore, this study can be viewed as an extension of traditional intercomparisons with prescribed large‐scale forcing (e.g., TOGA COARE and DYNAMO) and intercomparisons in which moisture source is defined as a relaxation to a prescribed profile [*Derbyshire et al*., 2004]. The results from this intercomparison project are important for understanding the two‐way interaction between convection and large‐scale tropical dynamics and also for interpreting discrepancies between the results reported in the literature. Our results suggested that the discrepancies between the published results can be related to the choice of the large‐scale parameterization method. For instance, we found that an individual model can produce different equilibrium states depending on the large‐scale parameterization method used.

Moreover, we found that even with exactly the same implementation of the WTG or DGW method, different SCM and even CRM models produce different sensitivities of the equilibrium state to SST. CRMs that participated in this study differ in their representation of subgrid‐scale processes that are important for the evolution of convection and its interaction with large‐scale circulation (e.g., cloud microphysics). The differences in CRMs lead to some diversity of behavior in RCE simulations [*Daleu et al*., 2015], and the diversity of behavior can be amplified when the physics is allowed to interact with the large‐scale dynamics. However, despite the diversity obtained among CRMs, our study demonstrates much larger intermodel variability among SCMs. That is, despite the significant differences in CRMs (e.g., resolution, domain size, microphysics, etc.), the behavior of these simulations using models with explicit convection are more constrained than those with parameterized convection.

This study has evaluated CRM and SCM sensitivities to parameterized large‐scale dynamical feedback with fixed radiation and a noninteractive surface. Further study may compare models and large‐scale parameterization methods with interactive radiation and/or an interactive surface. Since our study indicates that there is a greater consistency in the behavior of CRMs under parameterized large‐scale circulation while SCMs produce a much larger variation of behaviors, comparison between CRM and SCM behavior under parameterized large‐scale circulation may be a useful tool when developing and testing parameterization schemes. Therefore, further analysis may be to assess the impact of changes in parameterization within a particular SCM.
